# Artificial colloids versus human albumin for the treatment of ovarian hyperstimulation syndrome: A retrospective cohort study

**DOI:** 10.18502/ijrm.v17i10.5287

**Published:** 2019-11-07

**Authors:** Tetsuji Minami, MPH, Hayato Yamana, MPH Ph.D., Daisuke Shigemi, MPH, Hiroki Matsui1 MPH, Kiyohide Fushimi, Ph.D., Hideo Yasunaga, Ph.D.

**Affiliations:** ^1^Department of Clinical Epidemiology and Health Economics, School of Public Health, The University of Tokyo, Tokyo, Japan.; ^2^Department of Health Policy and Informatics, Tokyo Medical and Dental University Graduate School of Medicine, Tokyo, Japan.; ^3^Center for Reproductive Medicine, Dokkyo Medical University Saitama Medical Center, Saitama, Japan.

**Keywords:** Ovarian hyperstimulation syndrome, Treatment, Colloid, Length of stay.

## Abstract

**Background:**

The optimal colloid solution for the treatment of ovarian hyperstimulation syndrome (OHSS) remains to be established.

**Objective:**

We aimed to compare artificial colloids (AC) with human albumin (HA) for the treatment of OHSS.

**Materials and Methods:**

In this retrospective cohort study, data for OHSS participants were collected from a national inpatient database in Japan. The participants received intravenous fluid management with AC (n = 156) or HA (n = 127). We compared the two groups in terms of the length of stay, development of post-treatment complications, and termination surgery.

**Results:**

In multivariable linear regression analyses for log-transformed length of stay with reference to the OHSS participants receiving AC, the regression coefficient (95% confidence interval) in participants receiving HA was 0.03 (-0.04-0.09, p = 0.42). Thromboembolism occurred in two participants in the HA group and three participants in the AC group. Two participants in the HA group suffered renal failure during hospitalization. No participants underwent termination surgery in the two groups.

**Conclusion:**

The present results showed comparable efficacy between AC and HA for the treatment of OHSS. There were no significant differences in post-treatment complications between the two groups.

## 1. Introduction

Ovarian hyperstimulation syndrome (OHSS) is an iatrogenic and potentially life-threatening complication that affects 1–14% of all assisted reproductive therapy cycles (1). Exogenous gonadotrophin administration for the induction of ovulation can lead to ovary enlargement and fluid shift from intravascular spaces to third spaces because of an increased capillary permeability (2). Mild OHSS is self-limiting and gradually resolves by the time of the next menstrual period in patients who do not conceive. Patients who develop severe OHSS experience dyspnea, acute renal failure, and thromboembolism (3).

In patients at risk, the prevention of OHSS is the best strategy. Conservative treatments aim to relieve symptoms and prevent complications of OHSS. Patients with severe OHSS need to be admitted to hospital and receive careful monitoring, frequent examination, thromboprophylaxis, surgical intervention, and intravenous fluid management (4).

The crystalloid solution should be administered immediately to correct intravascular volume and provide end-organ perfusion. However, excessive administration can contribute to worsening ascites or pleural effusion. The colloid solution is a feasible option for diuresis. The optimal colloid solution for the treatment of OHSS remains to be established. A few studies have compared the outcomes between artificial colloids (AC; hydroxyethyl starch [HES] and/or dextran) and human albumin (HA) in OHSS patients, with inconsistent results (5, 6). However, these studies were limited by their small sample sizes.

In the present study, we compared AC with HA in terms of hospital stay and the development of post-treatment complications, using a nationwide inpatient database in Japan.

## 2. Materials and Methods

### Data source

The present retrospective cohort study utilized the Diagnosis Procedure Combination database, comprising administrative claims data and discharge information for acute-care inpatients in Japan. The database contains the following information: age and conception; main diagnoses on admission, comorbidities on admission, and complications developed during hospitalization encoded with International Classification of Diseases and Related Health Problems Tenth Revision codes; admission history for OHSS; interventions, including paracentesis, auto transfusion of concentrated ultra-filtered ascetic fluid, and oxygen administration; solutions, blood products, and drugs administered; and the dates of admission, discharge, and medical interventions. The database does not contain any laboratory data or imaging examination findings.

### Sample selection

We collected data from the women who were admitted to the hospital with a diagnosis of OHSS from July 2010 to March 2016. We included participants who were administered crystalloid solutions followed by colloid solutions within two days of admission. We excluded women who: (i) were diagnosed with adnexal torsion, ovarian rupture, thromboembolism, or cerebral infarction on admission; (ii) received any surgical procedure, such as exploratory laparotomy or adnexectomy, within two days of admission; and (iii) received both AC and HA. We divided the eligible participants into an AC group and an HA group based on their treatment. AC were defined as HES, dextran, and mannitol. HA included 5% or 25% products.

### Participants' background characteristics and outcomes

Data for the following background characteristics were collected: age; conception; OHSS history; comorbidity of polycystic ovary syndrome (PCOS); paracentesis; auto transfusion of concentrated ultra-filtered ascetic fluid; dopamine combined therapy; and total dose of crystalloid solution within two days of admission. Participants with respiratory comorbidities and those requiring respiratory support within two days of admission were classified as severe OHSS according to the OHSS classification (7, 8). Respiratory comorbidities were defined as pleural effusion, pulmonary edema, and acute respiratory distress syndrome. Respiratory support was defined as oxygen administration, pleuracentesis, and mechanical ventilation. Participants who received treatments for ascites and/or pleural effusion were divided into a paracentesis/pleura centesis group and an autotransfusion of the concentrated ultra-filtered ascetic fluid group.

The primary outcome was the length of stay (LOS). The secondary outcomes were the development of post-treatment complications and termination surgery. The post-treatment complications included adnexal torsion, ovarian rupture, thromboembolism, cerebral infarction, and renal failure after admission.

### Ethical consideration

The study was approved by the Institutional Review Board and Ethics Committee of The University of Tokyo (3501). The requirement for informed consent was waived because of the anonymous nature of the data.

### Statistical analyses

In comparisons of baseline characteristics and outcomes between the groups, categorical data were compared by the Chi-squared test or Fisher's exact test, and continuous data were compared using the Mann-Whitney U-test or Student's *t*-tests, as appropriate. In multivariable analyses, age was categorized into ≤ 34 and > 35 yr, and LOS was log-transformed because of its non-normal distribution.

The present study was a retrospective study, and the treatment allocation was not random. Decisions on treatment options were made by individual physicians taking the background characteristics of their participants into account. Therefore, we used multivariable regression analyses for log-transformed LOS with adjustment for patient background characteristics, while also adjusting for within-hospital clustering using generalized estimating equations.

All statistical analyses were conducted using SPSS Statistics version 22.0 (Statistical Package for the Social Sciences, IBM Corp., Armonk, NY, USA). The threshold for significance was p < 0.05 in all statistical analyses.

## 3. Results

We identified 2,161 OHSS cases in the Diagnosis Procedure Combination database from July 2010 to March 2016. Among these women, 283 were eligible for the present study (AC group, n = 156; HA group, n = 127). The distributions of the participants' characteristics are shown in Table I and the participants' outcomes are shown in Table II.

As shown in Table I, there were significantly higher proportions of severe OHSS, dopamine

combined therapy, and paracentesis/pleura centesis in the HA group compared with the AC group. The number of younger age (≤ 34 yr) women were 103 and 88, in the AC group and the HA group, respectively. The total amount of crystalloid solution in the HA group was significantly larger than that in the AC group.

As indicated in Table II, LOS was shorter in the AC group compared with the HA group (9 days vs 11 days, p < 0.001). Adnexal torsion, ovarian rupture, and cerebral infarction did not occur in either group. Pulmonary embolism was not detected in the two participants with thromboembolism in the HA group but was detected in one of the three participants with thromboembolism in the AC group. The two participants in the HA group suffered renal failure during hospitalization. There were no significant differences in post-treatment complications between the two groups. No participants underwent termination surgery in either group.

The results of the multivariable linear regression analyses for the primary outcome are shown in Table III. Older age (> 35 yr), conception, treatments for ascites and/or pleural effusion, and dopamine combined therapy were associated with longer LOS. However, with reference to the participants in the AC group, the regression coefficient (95% confidence interval) in the HA group was 0.03 (-0.04-0.09).

**Table 1:** Patient background characteristics


**Variables**		**Artificial colloid (n = 156)**	**Human albumin (n = 127)**	**P-value**
Age (yr)*		32.7 ± 4.4	32.0 ± 4.1	0.36${}^{a}$
Severe OHSS**#		9 (5.8)	34 (27)	< 0.001${}^{b}$
OHSS history**		10 (6.4)	10 (7.9)	0.65${}^{b}$
Comorbidity of PCOS**		5 (3.2)	4 (3.1)	1.000${}^{c}$
Conception**		26 (17)	36 (28)	0.069${}^{b}$
Type of treatment for ascites and/or pleural effusion**				
	None	124 (80)	99 (78)	0.753${}^{b}$
	Paracentesis/ pleura centesis	5 (3.2)	11 (8.7)	0.048${}^{b}$
	Auto transfusion of concentrated ultra-filtered ascetic fluid	27 (17)	17 (13)	0.365${}^{b}$
	Dopamine combined therapy**	82 (53)	86 (68)	0.011${}^{b}$
Total dose of crystalloid within 2 days (/100 ml)***		20 (15-25)	25 (15-31.6)	< 0.001${}^{a}$
*Data presented as mean ± SD; **data presented as n (%); ***data presented as median (IQR)				
a = Mann-Whitney U-test; b = Chi-squared test; c = Fisher's exact test				
#Participants with respiratory comorbidities and those requiring respiratory support within two days of admission were classified as severe OHSS				
OHSS: Ovarian hyperstimulation syndrome PCOS: Polycystic ovarian syndrome IQR: Interquartile range				

**Table 2:** Participants' outcomes


	**Artificial colloid (n = 156)**	**Human albumin (n = 127)**	**P-value**
Post-treatment complications*			
	Adnexal torsion	0 (0.00)	0 (0.00)	
	Ovarian rupture	0 (0.00)	0 (0.00)	
	Cerebral infarction	0 (0.00)	0 (0.00)	
	Thromboembolism	2 (1.3)	3 (2.4)	0.67${}^{a}$
	Pulmonary embolism	0 (0.00)	1 (0.8)	0.45${}^{a}$
	Renal failure	0 (0.00)	2 (1.6)	0.20${}^{a}$
	Termination surgery*	0 (0.00)	0 (0.00)	
	Hospital stay**	9.0 (6-13)	11.0 (8-17)	< 0.001${}^{b}$
*Data presented as mean ± SD; **data presented as median (IQR); IQR: Interquartile range				
a = Fisher's exact test; b = Mann-Whitney U-test				

**Table 3:** Multivariable linear regression analyses with general estimation equations for log-transformed length of stay


**Variable**		**Regression coefficient**	**(95% CI)**	**P-value**
Age (years)				
	≤ 34	Reference		
	> 35	0.062	(0.009-0.12)	0.021
Severe		-0.008	(-0.12-0.11)	0.89
OHSS history		-0.001	(-0.003-0.06)	0.97
Comorbidity of PCOS		-0.086	(-0.024-0.068)	0.27
Conception		0.27	(0.19-0.35)	< 0.001
Treatments for ascites and/or pleural effusion			
	None	Reference		
	Paracentesis/pleuracentesis	0.17	(0.043-0.17)	0.006
	Autotransfusion of concentrated ultrafiltered ascetic fluid	0.11	(0.048-0.29)	0.001
Dopamine combined therapy		0.14	(0.086-0.19)	< 0.001
<Total dose of crystalloid within two days (by 100-ml increase)		0.002	(-0.001-0.004)	0.26
Treatment group			
	Artificial colloid	Reference		
	Human albumin	1.3	(-0.81-3.34)	0.23
CI: Confidence interval; OHSS: Ovarian hyperstimulation syndrome; PCOS: Polycystic ovarian syndrome				
Older age (> 35 yr), conception, treatments for ascites and/or pleural effusion, and dopamine combined therapy were associated with longer LOS. However, with reference to patients in the artificial colloid group, the regression coefficient (95% confidence interval) in the human albumin group was 0.03 (-0.04-0.09)				

## 4. Discussion

In the present large retrospective cohort study using a nationwide inpatient database in Japan, we found comparable efficacy between AC and HA for intravenous fluid management in OHSS participants, after adjustment for confounding factors. In addition, most of the OHSS participants admitted without critical comorbidities had uneventful courses without the development of post-treatment complications.

A small-sized retrospective study suggested that HES may be superior to HA in colloid solutions for reducing LOS and paracentesis, and increasing urine output (5). A non-randomized clinical trial suggested that dextran had greater efficacy for the treatment of hemoconcentration caused by OHSS than HA, but did not assess complications and LOS (6). Therefore, the optimal colloid solutions for the treatment of OHSS remain to be established, and the present study showed comparable efficacy between AC and HA. This finding is pathophysiologically plausible because these colloid solutions have no effect on the hypothalamic-pituitary-ovarian system, and are biochemical substances that increase capillary permeability and vascular endothelial cells. HA solution is a finite resource and should be supplied optimally to patients with severe diseases, such as liver cirrhosis, nephrotic syndrome, and hemorrhagic shock. Infections remain of concern, especially in reproductive-age women. In particular, human parvovirus B19 infection by the administration of blood products can lead to miscarriage, fetal edema, and anemia (9). In addition, HA is 10-fold as expensive as AC (approximately $45 per 5% albumin 250 ml vs $4.5 per AC 250 ml) (10).

AC has several advantages in terms of being biohazard-free, cost-effective, and safe. It is conceivable that AC may have adverse effects on renal function. Although a randomized clinical trial showed no evidence for a colloid-related increase in the risk of renal replacement therapy among hypovolemic participants in the intensive care unit (11), the importance of compliance with the dose recommended by regulatory agencies was mentioned. We considered that the results of the previous study could be generalizable to the OHSS participants. However, there were several concerns about the aggressive use of AC. First, HES and dextran can interact with the blood coagulation system. In one case report involving OHSS, this interaction led to delayed persistent pleural effusion and significant blood coagulation alteration despite hormonal resolution (12). Meanwhile, this interaction can potentially be used for thromboprophylaxis (13). Second, there may be effects on fetuses. Two studies showed that HES did not cross the placental barrier in rats and sheep (14, 15). Although these findings may also apply to humans, further studies are required.

Late-onset OHSS, caused by conception, is correlated with endogenous gonadotrophin produced by an embryo or administration of gonadotrophin for luteal phase support (16). The present results are consistent with the fact that endogenous gonadotrophin can lead to worsening and prolongation of OHSS.

Longer LOS was significantly associated with treatments for ascites and/or pleural effusion and dopamine combined therapy but was not associated with severe OHSS. Our classification of severe OHSS depended on respiratory comorbidities and the requirement for respiratory support, and thus we could have misclassified severe OHSS participants who received treatments for ascites and/or pleural effusion and dopamine combined therapy.

Several studies showed that younger age, OHSS history, and comorbidity of PCOS were risk factors for developing OHSS (8, 16-18). In the present study, these factors were not associated with LOS. A possible explanation for these findings is that physicians may have tended to admit such high-risk participants for even mild OHSS.

We did not assess allergic reactions because recorded diagnoses of allergic reactions may not be well validated due to the retrospective nature of the data. A previous systematic review showed little or no difference in allergic reactions for the use of AC vs HA (19).

### Limitations

Several limitations should be addressed. First, data on precise body mass index were not available, because OHSS participants may have been overweight because of ascites. The database did not include data on artificial reproductive technologies including ovulation induction, presence or absence of luteal phase support, single or multiple embryo transfers, and ovarian reserve markers. Although the associations of these components with OHSS development were reported (16, 20), the associations with OHSS prolongation have not been reported. Second, the study was not a randomized clinical trial, and possible unmeasured confounders may have affected the results. Third, because the Diagnosis Procedure Combination database was not created for research purposes, the findings should be interpreted carefully, because recorded diagnoses in retrospective databases are generally less well validated than those in planned prospective studies. However, a previous validation study of the Diagnosis Procedure Combination database showed high specificity of diagnoses and procedure records (21). Finally, we could not follow the maternal and neonatal outcomes, especially miscarriages that did not require interventions and live birth rates.

## 5. Conclusion

In conclusion, our results showed comparable efficacy between AC and HA for intravenous fluid management in OHSS patients. We suggest that the use of AC was optimal in terms of their biohazard-free nature, cost-effectiveness, and safety.

##  Conflict of Interest

No conflicts of interest are disclosed.
